# Numerical Modeling of Interstitial Fluid Flow Coupled with Blood Flow through a Remodeled Solid Tumor Microvascular Network

**DOI:** 10.1371/journal.pone.0067025

**Published:** 2013-06-26

**Authors:** M. Soltani, P. Chen

**Affiliations:** Waterloo Institute for Nanotechnology, Department of Chemical Engineering, University of Waterloo, Waterloo, Ontario, Canada; University of Adelaide, Australia

## Abstract

Modeling of interstitial fluid flow involves processes such as fluid diffusion, convective transport in extracellular matrix, and extravasation from blood vessels. To date, majority of microvascular flow modeling has been done at different levels and scales mostly on simple tumor shapes with their capillaries. However, with our proposed numerical model, more complex and realistic tumor shapes and capillary networks can be studied. Both blood flow through a capillary network, which is induced by a solid tumor, and fluid flow in tumor’s surrounding tissue are formulated. First, governing equations of angiogenesis are implemented to specify the different domains for the network and interstitium. Then, governing equations for flow modeling are introduced for different domains. The conservation laws for mass and momentum (including continuity equation, Darcy’s law for tissue, and simplified Navier–Stokes equation for blood flow through capillaries) are used for simulating interstitial and intravascular flows and Starling’s law is used for closing this system of equations and coupling the intravascular and extravascular flows. This is the first study of flow modeling in solid tumors to naturalistically couple intravascular and extravascular flow through a network. This network is generated by sprouting angiogenesis and consisting of one parent vessel connected to the network while taking into account the non-continuous behavior of blood, adaptability of capillary diameter to hemodynamics and metabolic stimuli, non-Newtonian blood flow, and phase separation of blood flow in capillary bifurcation. The incorporation of the outlined components beyond the previous models provides a more realistic prediction of interstitial fluid flow pattern in solid tumors and surrounding tissues. Results predict higher interstitial pressure, almost two times, for realistic model compared to the simplified model.

## Introduction

Although heart and cardiovascular diseases were the primary causes of death for several decades, more than 12 million new cancer cases were reported around the world in 2011, making it the new leading cause of death. More than 85% of cancer incidents involve solid tumors. The condition of the disease aggravates or the treatment process becomes inefficient as the drug delivery to the present solid tumors is usually incomplete. In fact, the main barrier to successful drug delivery to solid tumors is their abnormal and complicated vasculature [1]. The high interstitial pressure and low intravascular pressure near the tumor affect drug transport, causing slow flow through the tissue and low filtration of drugs from vessels. Therefore, better understanding of tumor formation is crucial in developing more effective therapeutics [2]. For this purpose, nowadays, solid tumor modeling and simulation results are used to predict how therapeutic drugs are transported to tumor cells by blood flow through capillaries and tissues. This facilitates identifying better methods for delivering targeted anticancer therapies [3], [4].

**Figure 1:**
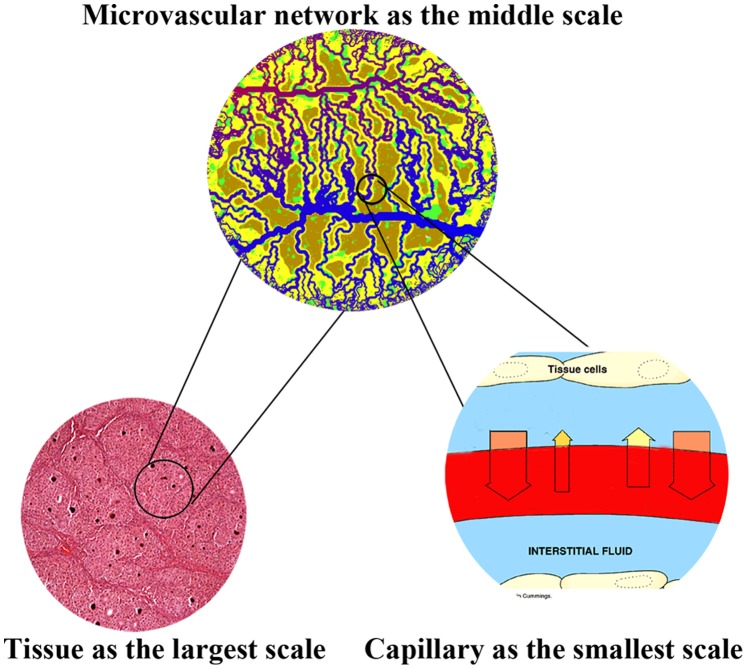
Schematic of different scales of simulated solid tumor growth.

Solid tumor growth can be characterized in two different phases: avascular and vascular. Relying only on diffusion from nearby vessels to supply oxygen and nutrients, the first phase usually continues until a tumor reaches the diameter of a few millimeters [5]. To grow further, a solid tumor needs its own blood supply system, in the form of, and supplied by, a capillary network, which is deemed as the emergent of second phase. Both of these are complicated processes which take place in a wide range of spatial and temporal scales. This multi-scale nature of tumor formation makes the governing equations involved in the mathematical modeling highly complex to solve. Multi-scale modeling involves: the convection and diffusion of fluid flow in normal and tumor tissues at the largest scale, blood flow distribution through a network generated by tumor-induced angiogenesis at the middle scale, and blood flow convection in capillaries by considering non-continuous behavior of blood and adaption of capillary diameter at the smallest scale (Figure 1).

Modeling of diffusion and convection of fluid flow is common in solid tumor tissue while considering the domain to be a porous medium with a uniform source and sink of blood flow. Maintaining this consideration, Jain et al. [6]–[8] used a model of a spherical tumor that had continuously distributed vasculature in the presence of the lymphatic system. The main assumption used to calculate pressure distribution in their model is that the net flow into the interstitial space from the vasculature is balanced by the efflux to the lymphatic system through Starling’s law.

In the model proposed by Netti et al. [9], tumor vasculature was treated as an equivalent permeable vessel embedded in a uniform-pressure medium. Simulations were used to examine the effects of vessel leakiness and compliance, as well as the interstitial fluid pressure. Soltani et al. [2] developed a mathematical model of interstitial fluid flow using a numerical element-based finite volume method for modeling the continuity equation in the porous media of spherical tumors. They introduced two new parameters: the critical tumor radius and critical necrotic radius. They also applied their model to different geometries of tumors to study the effects of tumor shape and size in drug delivery [10]. None of these earlier works considered the effects of the tumor-induced network generated by tumor angiogenesis on fluid flow in normal and tumor tissues. In these works, in order to have reasonable results comparable to or verifiable by experimental data, a reasonable constant estimation with uniform distribution is made for intravascular pressure.

Stephanou et al. [11] have examined flow modeling in a tumor-induced capillary network. They used continuous and discrete mathematical models of angiogenesis, described by Anderson and Chaplain [12], for generating a capillary network. The continuous model uses differential equations and is based on the mass conservation equation and chemical kinetics [12]. The discrete method of angiogenesis modeling uses a similar set of equations as that of the continuous model but with different interpretation of the coefficients, movement probabilities [12]. This method is capable of modeling the growth and motility of endothelial cells. Thus Stephanou et al. [11] performed a Newtonian fluid flow simulation through 2D and 3D rigid capillary networks in order to investigate chemotherapy treatment efficiency. Wu et al. [13] presented a numerical model that combines intravascular, transvascular, and interstitial fluid movements in 3D capillary networks originating from tumor-induced angiogenesis. The mentioned models do not take into account the non-Newtonian rheological nature of blood in capillaries or remodeling of microvessels in tumor-induced networks.

Blood is a complex fluid and blood flow study in different sizes of vessels shows that blood has non-Newtonian behavior [14], [15] and has interactions with vessel’s wall even in large vessels [16]. Small size of capillaries compared to the size of red blood cells causes a non-continuous blood flow. The non-continuous behavior of blood includes variation of blood viscosity with tube diameter and hematocrit – the fraction of the total volume of blood occupied by red blood cells – and an unequal fraction of hematocrit between the branches of capillary bifurcation. The capillaries adapt their diameter based on signals received from hemodynamic stimuli such as wall shear stress, pressure, and metabolic processes. The non-continuous behavior of blood in capillaries and structural adaptation of the vasculature has also been incorporated into models including capillary networks [17]–[19]. Owen et al [17] developed a multi-scale model of vascular tumor growth linking vascular adaptation, blood flow, oxygen, and growth factor transport at the tissue scale to the subcellular and cellular dynamics of normal and cancerous cells. In their most recent study [20], they extended the work to a 3D model.

Stephanou et al. [18] modeled an adaptive network associated with tumor-induced angiogenesis and considered the effect of phase separation of blood flow in bifurcation and non-Newtonian behavior of blood. They investigated the effects of this remodeling on drug delivery to tumor cells. McDougall et al. [19] introduced several improvements in a model presented in [18], by examining the flow of a non-Newtonian fluid in a dynamic adaptive network. They developed a mathematical model that simultaneously combines vessel growth with blood flow through a capillary. In the mentioned works, only intravascular blood flow was studied and for the sake of simplicity the intravascular flow was considered to be independent from the interstitial flow surrounding the capillaries.

Wu et al. [21] developed a mathematical model of tumor microcirculation, which coupled microvasculature and interstitial space perfusion, and combined intravascular and interstitial flow by vascular permeability. In their work, only the vessels located inside the tumor region were considered to adapt their diameter based on the compliance method presented by Netti et al. [9]. In the adaptation method used by Wu et al. [21], the effects of hemodynamic and metabolic stimuli were not considered, and their compliance method was implemented only in vessels inside the tumor tissue; normal tissue vessels were assumed to have a constant diameter.

In spite of the valuable body of work performed in simulation of fluid flow in normal and tumor tissues, previous studies have not examined the interaction of interstitial flow through tissue and intravascular flow through adaptive capillary network. To address this shortcoming, this work introduces a mathematical model that simultaneously couples interstitial fluid flow with convective non-continuous blood flow through vessels by considering a remodeling network based on hemodynamic and metabolic stimuli. The other significance of presented method is calculating intravascular pressure distribution and values in tumor and normal tissues instead of assuming constant pressure.

This paper describes a non-continuous blood flow model through a capillary network induced by a tumor combined with interstitial flow in normal and tumor tissues in 2D, taking into account the extravasation flux of fluid across the vasculature. First, the governing equations of angiogenesis are implemented to specify a capillary network in the interstitial domain. A discrete method initially presented by Anderson and Chaplain [12] is used to create the vascular network in both normal and cancerous tissues. The governing equations are then introduced for blood flow through the capillary network and fluid flow in interstitium. Finally, the effect of the capillary network on interstitial fluid flow is investigated, considering three approaches for simulating blood flow in the network; 1) the governing equation for interstitium is solved without considering capillary network, as in [2], [8], [10]; 2) similar to the approach used in [11], [13], the tumor-induced network is presumed to have rigid capillaries, and blood flow is calculated in the network by mass conservation at each junction; 3) the capillaries are assumed to adapt their diameter in response to signals received from metabolic and hemodynamic stimuli. The remodeling method presented by Pries et al. [22] is used for formulating capillary adaptation. For this reason, phase separation, based on the method presented by Fung, is involved in the third approach [23] and also non-Newtonian behavior of blood, based on the formula presented by Pries et al. [24], is implemented. Results show that fluid flow modeling in normal and tumor tissues with a vascular network added by non-continuous behavior of blood and adaptability of capillary predicts higher pressure levels compared to previous work on interstitial flow [2]. Also intravascular pressure distributions show that considering uniform distribution for pressure is not a reasonable assumption as may result in totally different values for intravascular pressure.

## Mathematical Model of Angiogenesis

**Figure 2:**
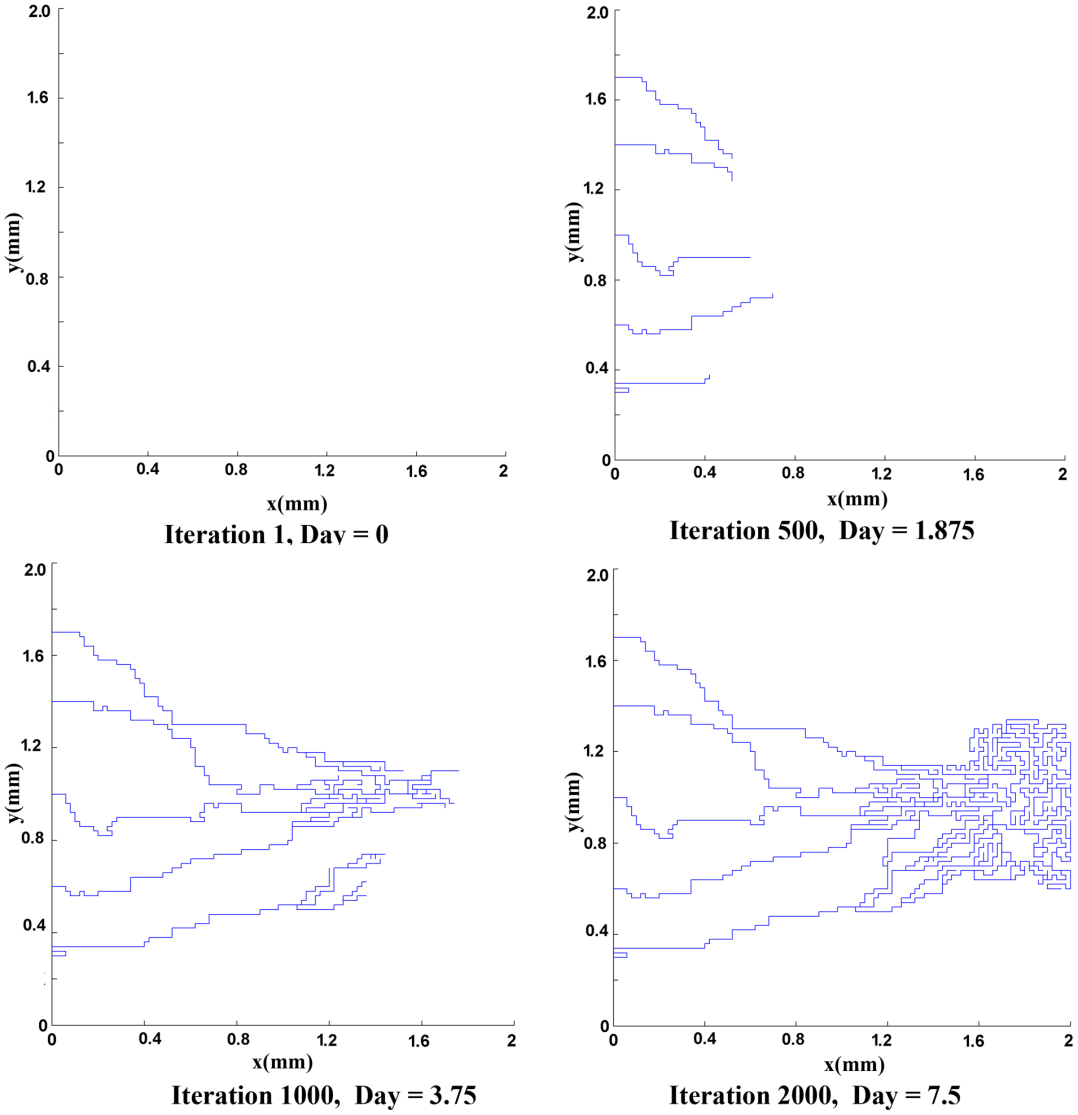
The results of discrete sprouting angiogenesis. Five initial sprouts move toward the tumor on the right of the domain.

**Figure 3:**
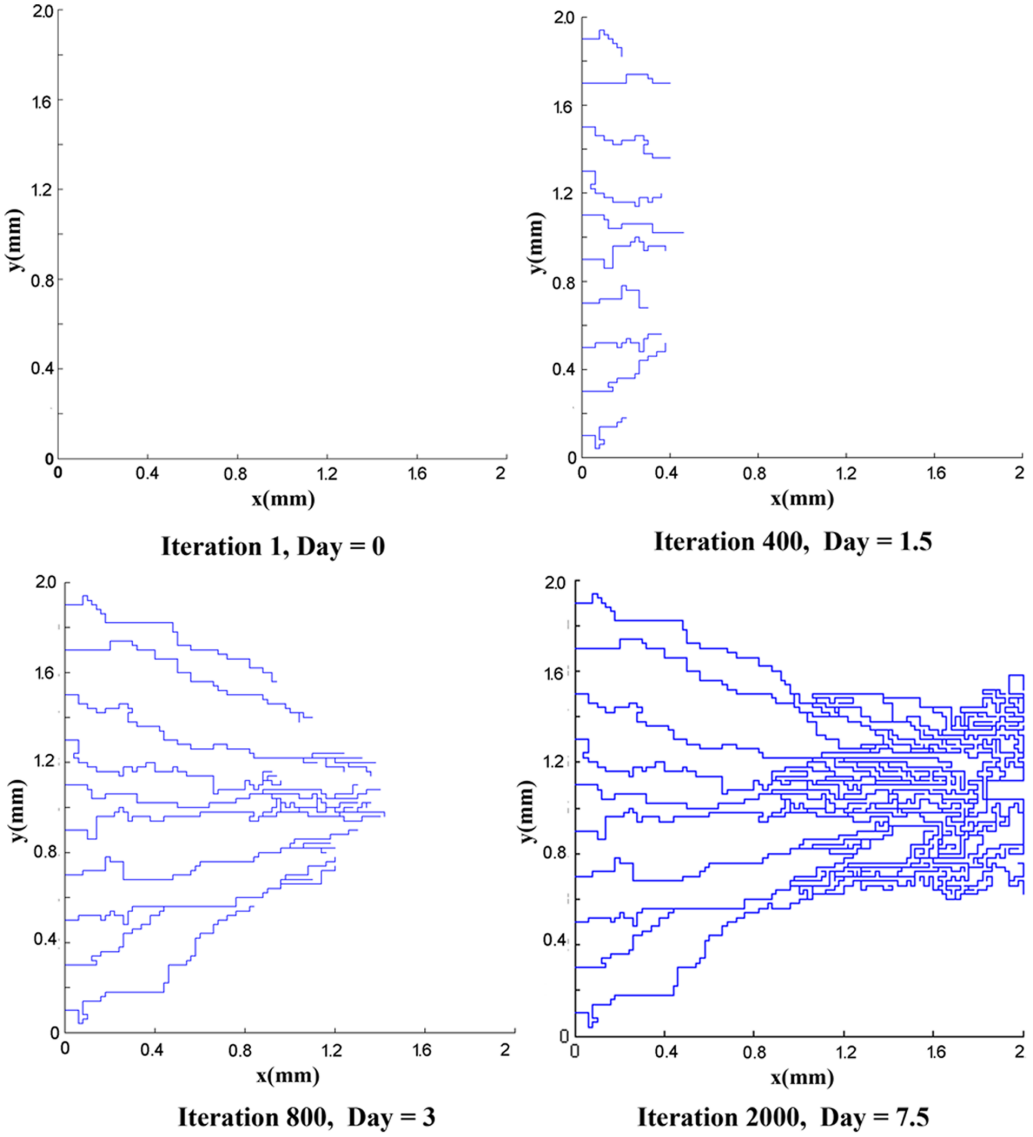
The results of discrete sprouting angiogenesis. Ten initial sprouts move toward the tumor on the right of the domain.

The mathematical model used for generation of the network is based on the work of Anderson and co-workers [12]. To generate the networks, two initial conditions for the number of endothelial cells in the parent vessel are considered: 5 endothelial cells and 10 endothelial cells. Results of capillary network formation are shown in Figures (2) and (3). These networks are used for further studies in the next section.

### Flow Simulation in Capillary Networks

The analysis of blood flow in capillary networks is generally the same as the analysis of other networks, but with a few significant differences. These differences are due to the permeability of vessels, changeable diameters of vessels, non-continuous behavior of blood in micro-scale vessels, and the porous nature of the medium surrounding the vessels. In this section, the governing equations for a network and porous media are presented. Then the models used for the non-continuous behavior of blood and vessel adaptation are introduced.

**Figure 4:**
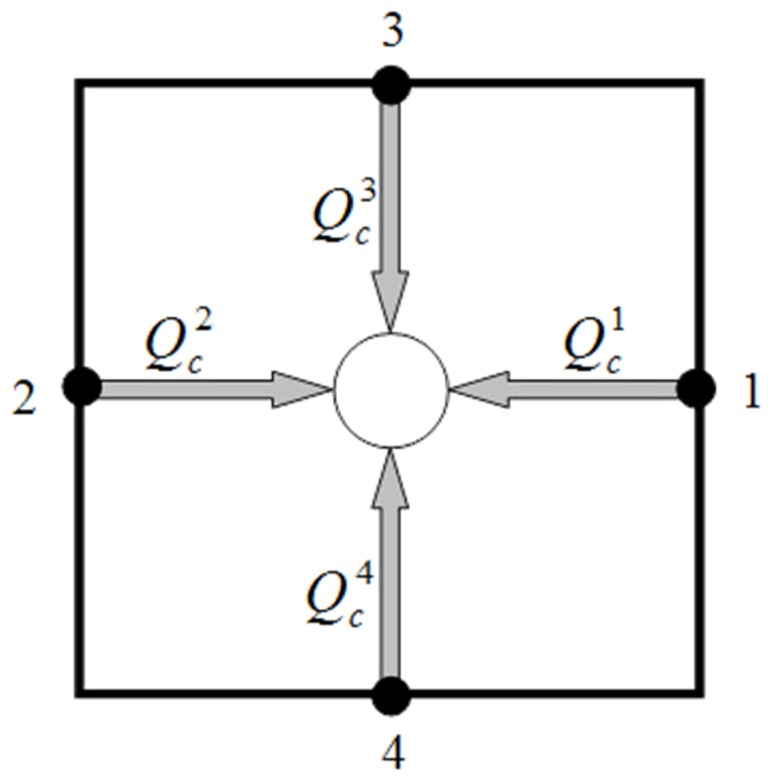
Schematic representation of blood flux at each vascular node.


**Figure 5:**
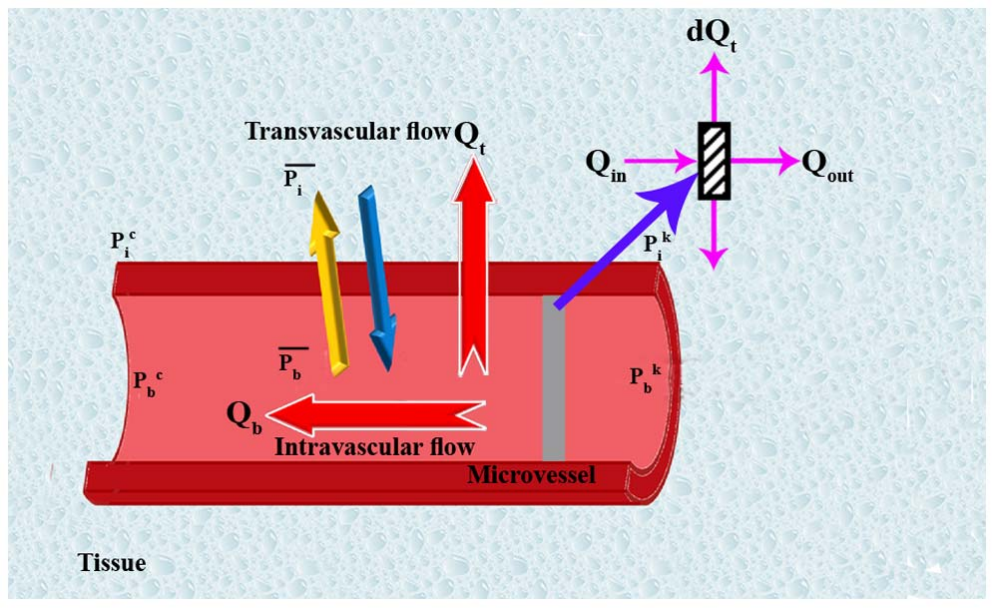
Schematic representation of blood flux through capillary or transformation from capillary and related parameters.


A network of blood vessels and electrical resistors are very similar. Pressure and electrical potential play the same role in this analogy. Volume flow rate and electrical current have similar roles as well. Wong et al [14], [15] used this analogy for mathematical modeling of coronary arteries with atherosclerosis and provided a simple, elegant, non-invasive, and optimum method in terms of computational cost to predict flow properties for geometrically complex pathology at micro-scale levels. By assuming that the rheological parameters are known, the numerical method – a linear analysis – is utilized in calculating the flow rate in each element and pressure value at each node. The flow rate in each vessel is calculated by applying mass (or volumetric flow rate) conservation law at each junction of the network. The equation representing the volumetric flow rate for an interconnecting point like c (Figure 4) in the network can be written as
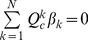
(1)where the index *k* refers to adjacent nodes and *N* is the number of adjacent nodes. In 2D simulation for a fully connected network, *N* is 4, and 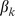 is a positive integer ‘1′ or ‘0′, which describes whether nodes c and k are connected (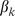 = 1) or not connected (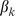 = 0).

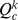
 is the net flow rate for each capillary and includes the flow through the capillary and transvascular flow from each capillary. As shown in Figure (5), 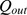 is net flow rate and related to 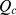, and 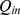 is inlet flow and related to Poiseuille flow, 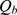. By implementing mass conservation law to an element of a vessel, we have
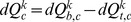
(2)where

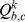
 is the blood flow through each capillary, and


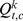
 is the transvascular flow (the extravasation fluid flux from the vessels).

Based on Equation (1), when the calculated value of the flow rate is positive, the flow comes into the node and when it is negative, the flow goes out of the node.

The transvascular flow rate is calculated by Starling’s law, which represents the role of hydrostatic and oncotic pressures in the movement of fluid across capillary membranes. Starling’s law [23], [25] is calculated by.
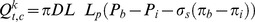
(3)where


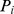
 is the interstitial pressure,


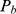
 is the intravascular pressure,


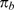
 is the osmotic pressure of the plasma,


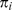
 is the osmotic pressure of the interstitial fluid,


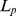
 is the hydraulic conductivity of the vessel wall,


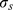
 is the average osmotic reflection coefficient for the plasma proteins.

D is the vessel diameter, and

L is the vessel length.

By substituting Equation (3) into Equation (2), the differential equation is obtained as follows
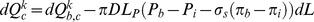
(4)


The solution of the differential equation is mentioned by Pozrikidis in [4]. For sake of simplicity, Pozrikidis considered that the 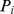 is constant for decoupling it from the interstitial flow and solving it in isolation. Also he assumed that the vessel diameter is constant during the simulation. The solution leads to an integral equation. In our work, the interstitial pressure and vessel diameter changes are considered. For simplicity, we considered the average of Pb and Pi in Starling’s Law for each segment. Therefore, integrating both sides of the Equation (4) results in
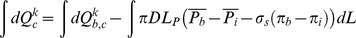
(5)

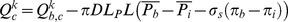
(6)where


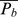
 is the average blood pressure in vessels, calculated by 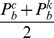, and


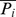
 is the average interstitial fluid pressure outside of the vascular element, calculated by 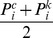.

Blood flow in capillary tubes has low Reynolds number, much less than 1. For such a low Reynolds number, Poiseuille’s law can be applied. Poiseuille’s experiment shows the relationship between the flow rate 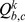 and the driving pressure ΔP in a tube of diameter D and length L. Theoretical analysis leads to the following equation:
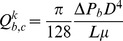
(7)where

Δ*P_b_* is calculated by 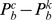, and 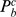 and 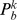 are corresponding blood pressures at each node.

By substituting Equation (7) into Equation (6):
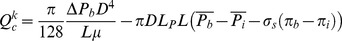
(8)


Calculation of transvascular flow rate depends on intravascular pressure in capillaries and interstitial pressure in normal and tumor tissues. The intravascular pressure is found by solving the mass conservation equation in the network by applying Poiseuille’s equation for flow in capillaries as mentioned. The interstitial pressure for peripheral tissue of a vascular network is found by solving the governing equation for fluid flow through a porous medium. In fact, Equation (3) couples blood flow in the network and fluid transport in the tissue around the network.

Normal and tumor tissues have properties like those of a porous medium. One of the earliest formulations for flow transport in porous media is Darcy’s law. Darcy’s empirical observations show that the fluid velocity in porous media is proportional to the pressure gradient; therefore, fluid transport in the porous media can be described by the following equation [7], [23]:
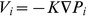
(9)where


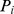
 is the interstitial pressure,


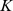
 is the hydraulic conductivity of the interstitium,


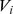
 is the interstitial fluid velocity.

The mass balance equation for a steady state incompressible fluid shows that the divergence of the velocity is zero, or mathematically,
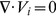
(10)


This equation is acceptable for porous media when there is no fluid source or sink in the medium, but biological tissues have sources and sinks. For instance, between the interstitial space and the blood or lymph vessels, fluid is exchanged; therefore, the steady state incompressible form of the continuity equation must be modified as in [2]

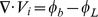
(11)where


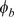
 is the rate of fluid flow per unit volume from blood vessels into the interstitial space, and.


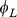
 is the rate of fluid flow per unit volume from the interstitial space into lymph vessels.

It should be noted that Equation (11) in its general form is applicable to any kind of biological tissue, whether normal or cancerous. In this simulation, the value for lymph vessels is neglected. The blood vessel terms (or fluid source terms) can be evaluated through Starling’s law as follows:
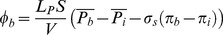
(12)where 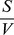 is the surface area per unit volume of tissue for transport in the interstitium. Other terms in Equation (12) were introduced previously in this section.

The mass conservation equation is obtained for normal and tumor tissues with a capillary network by adding a source term for mass flow to the right-hand side of the continuity equation wherever there is a capillary; otherwise, the right-hand side of the continuity equation is simply zero, or mathematically
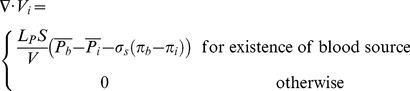
(13)


**Figure 6:**
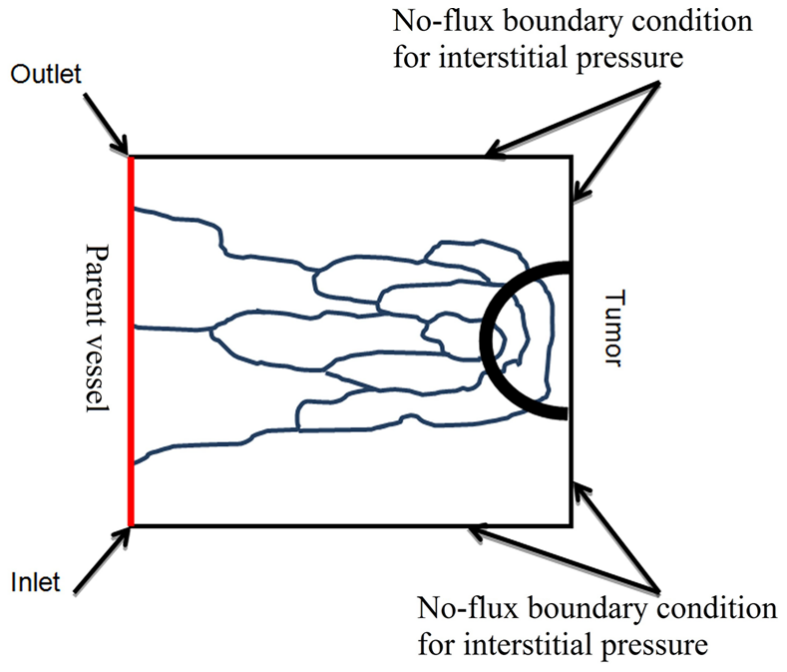
A schematic of calculated domain for fluid flow simulation.

Combination of the continuity equation and Darcy’s law results in
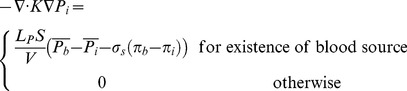
(14)when hydraulic conductivity, *K*, is constant:



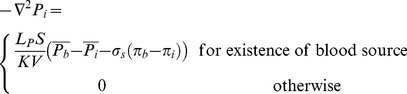
(15)Applying the appropriate boundary conditions and also using all the constants mentioned in Table (1), the governing equation, Equation (15), can be used to calculate interstitial fluid pressure in both normal and tumor tissues. As shown in Figure (6), a no-flux boundary condition is considered for the right-hand side of the domain and upper and lower limits of the domain, i.e.,
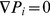
(16)


**Table 1:** Material properties used in numerical simulations, as taken from [2].

Parameter	Normal Tissue	Tumor Tissue
L_p_[cm/mmHg s]	0.36×10^−7^	2.80×10^−7^
K[cm^2^/mmHg s]	8.53×10^−9^	4.13×10^−8^
S/V[cm^−1^]	70	200
π_B_[mmHg]	20	20
π_i_[mmHg]	10	15
σ	0.91	0.82

For the left-hand side of the domain near the parent vessel, the constant value of pressure is considered.

Equation (1), by considering Equation (8), and Equation (15), should be solved simultaneously to find the intravascular, P_b_, and interstitial, P_i_, pressures.

The solution can be obtained numerically to find intravascular pressure and interstitial pressure and other related parameters such as velocity and flow rate. The iterative numerical method, Gauss–Seidel method [26], is applied to solve the system of equations for intravascular pressure. A finite difference method is applied to discretize Equation (15). The discretized form of the governing equations, in their general form, is then linearized and solved explicitly by an iterative procedure. This iterative method can be called semi-implicit, as during the solution, the most updated values of pressures are used. The algorithm for the procedure and discretized form of the equations are described in Section 4.

### 3.1 Blood Viscosity in Capillaries

As mentioned, Poiseuille’s law can be used for Newtonian flow, but blood has significant non-Newtonian properties at low Reynolds numbers. Blood viscosity in capillaries depends on the vessel diameter and hematocrit. To take advantage of Poiseuille’s law’s simplicity and use it to show the behavior of blood, it is helpful to define the apparent or effective viscosity of blood, according to Equation (7), by.
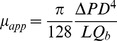
(17)


Pries et al. [24], [27] used data obtained from the results of 18 studies of human blood and combined the data with a parametric description of apparent blood viscosity relative to the plasma viscosity to define a mathematical function for apparent viscosity. They introduced this parameter as the relative apparent viscosity. A description of relative apparent viscosity as function of the tube diameter and hematocrit is as follows [24]:
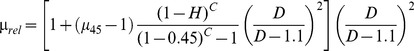
(18)µ_45_, the relative apparent blood viscosity for a fixed hematocrit of 0.45, is given by.
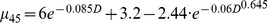
(19)where

*D* is the vessel diameter (in µm),

*C* describes the shape of viscosity dependency on the hematocrit, defined as
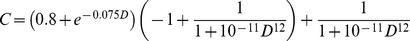
(20)and



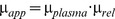
(21)The two parameters, hematocrit and vessel diameter, affecting blood viscosity are dependent on blood flow characteristics such as velocity, wall shear stress, and pressure in vessels. These dependencies are described in the next sections.

### 3.2 Phase Separation at Capillary Bifurcations

**Figure 7:**
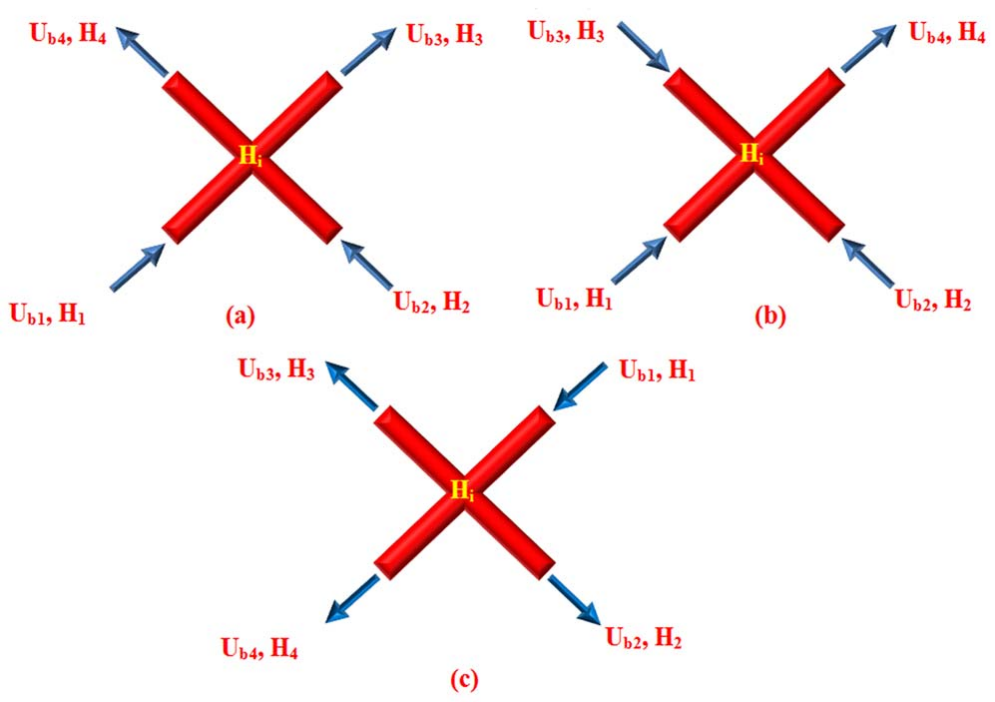
Schematic of different patterns of blood flow in networks a) Blood flow from one (two) node(s) into one (two) node(s). b) Blood flow from three nodes into one node. c) Blood flow from one node into three nodes.

Since µ_rel_ depends on H, the hematocrit distribution has a significant role in simulating the hemodynamic characteristic of the capillary network. In general, H distribution at a vessel bifurcation depends on the flow velocity in each branch. The total red blood cell fraction in the feeding vessel of a bifurcation that goes into one of the daughter branches is not essentially the same as the fractional blood flow going into that branch. It has been shown that all the hematocrit goes into the branch with the faster velocity at bifurcations if the velocity at that branch goes beyond a certain limit. This phenomenon – also referred to as plasma skimming – was observed early on by Krogh to describe the skimming of the cell-poor marginal fluid layers of an arteriole feeding vessel by its smaller side branch [28]. Three related experimental evidences support this model [29]: (i) The red cells are not in general distributed proportionally to blood volume flow, unless the hematocrit in the daughter vessels differs from that of the inflow vessel. (ii) When blood flow is divided equally between the daughter vessels, the red cell flow is not distributed evenly. (iii) There is a critical fractional blood flow to a daughter vessel below which it receives no red cells. Fung [23] proposed a theoretical model to describe this phenomenon. Extending Fung’s conclusions [23] results in the distribution rules of H, as follow (see Figure 7) for definitions, assuming U_b2_≥U_b3_≥U_b4_) [30]:

a) Blood flow from one (two) node (s) into one (two) node (s) (Figure 7a).
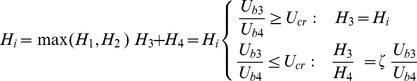
(22)


b) Blood flow from three nodes into one node (Figure 7b)
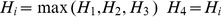
(23)


c) Blood flow from one node into three nodes (Figure 7c)
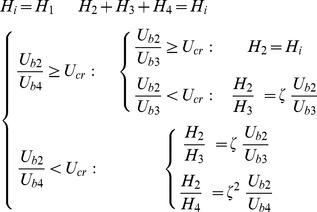
(24)where H_i_ denotes H at node i, ζ is a phenomenological parameter that accounts for the strength of the non-symmetry of the hematocrit distribution at bifurcations [30], and U_cr_ is the critical ratio of the velocities of the branches, above which, the total hematocrit goes to the faster branch [30]. Here, the hematocrit in the parent vessels and their joint nodes with the connected induced network is assumed to be a constant 0.45.

### 3.3 Vessel Diameter Adaptation

Capillaries are able to continuously adapt their diameter in response to the functional requirements of the tissues that capillaries supply [22]. Each vessel responds locally to physical and biochemical stimuli. In fact, wall shear stress and intravascular pressure created by blood flow lead to remodeling of the vessel diameter. The biochemical stimuli such as the metabolic stimulus are also able to remodel the capillary network. For each vessel in the network, the change of its diameter (Δ*D*) for a time step (Δ*t*) is assumed to be proportional to the stimulus term, its initial diameter, and the time step [22]:
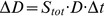
(25)


S_tot_ includes the influences of the wall shear stress (S_wss_), the intravascular pressure (S_p_), and a metabolic mechanism depending on the blood hematocrit (S_m_). Each stimulus is next briefly discussed.

#### 3.3.1 Wall shear stress

Experimental observations show that to maintain a specific level of wall shear stress, the vessels adapt their diameter. Murray [31] showed that when the volumetric blood flow rate in each vessel is proportional to the cube of the capillary diameter, the energy consumption for pumping the blood to the circulatory systems is minimal. The wall shear stress in a capillary vessel is given by
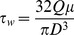
(26)


Based on Equation (26), to satisfy Murray's law, wall shear stress needs to be approximately constant in vessels [32]; therefore, when each vessel senses wall shear stress, it adjusts its diameter to achieve a uniform level of stress. Experimental observation [22] shows that vessel diameter increases when wall shear stress increases. This stimulus is described by the following logarithmic law:
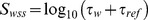
(27)where 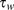 is the actual wall shear stress in a vessel segment calculated by Equation (26), and 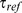 is a small constant included to avoid singular behavior at low wall shear stress values.

#### 3.3.2 Intravascular pressure

**Figure 8:**
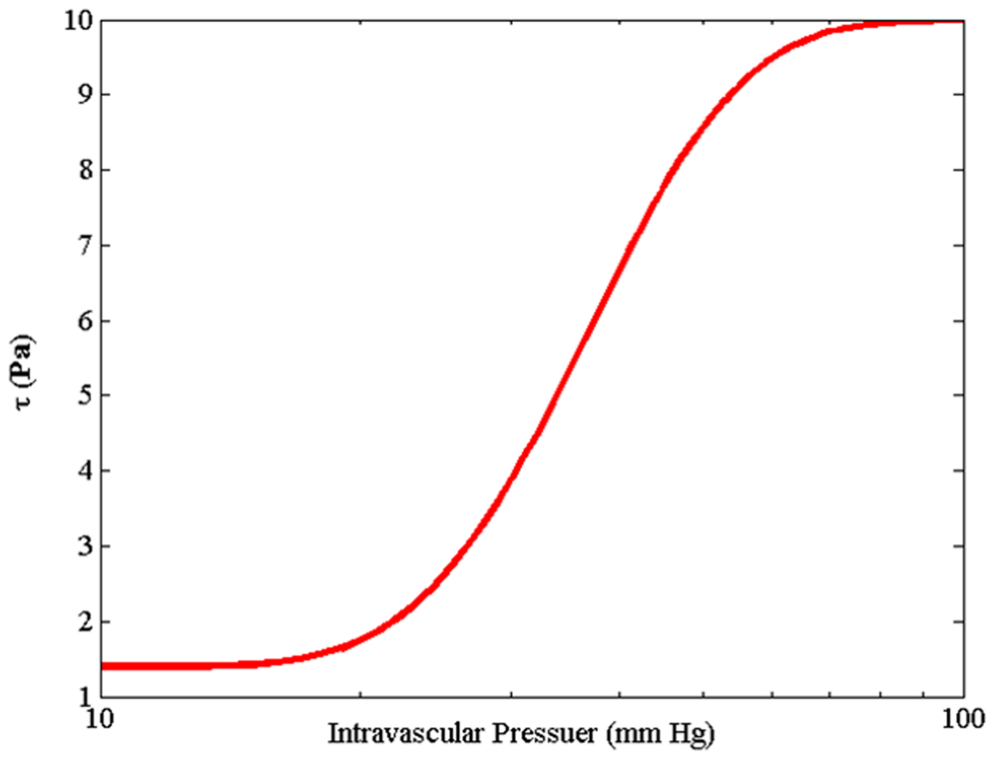
Shear stress induced by intravascular pressure in a range of intravascular pressure flows in circulatory system. Equation is derived by Pries et al. [33] with curve fitting from experimental results obtained on rat mesentery vessels.



Shear stress values differ significantly between arterial and venous vessels. The values in the arterial system are always greater than those in a venous one [22]. Studying structural adaptation, Pries et al, suggested a preset relationship between local intravascular pressure and wall shear stress [33]. They showed that increasing intravascular pressure decreases vessel diameter. To consider the corresponding stimulus of intravascular pressure, they suggested a negative sensitivity represented by the following equation:
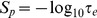
(28)in which 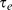 is the wall shear stress resulting from the blood pressure, P_b_, based on the experimental data on rat mesenteries [33]. As shown in Figure (8), this wall shear stress increases, in a sigmoidal shape, as pressure increases.

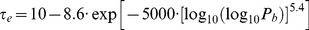
(29)In this equation, pressure and stress are obtained in mmHg and Pa, respectively. As shown in Figure (8), for pressure less than 10 mmHg, Equation (29) is not applicable. Instead, the value of 1.4 Pa is considered.

#### 3.3.3 Metabolic hematocrit-related stimulus

The metabolic requirement of tissue can affect and control the vessel diameter. A drop in oxygen supply or other metabolic material stimulates the vessel to increase its diameter to enhance perfusion. Pries et al. [34] suggested adding a stimulus dependency to the volumetric flux of red blood cells passing through a vessel (represented by 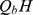), thus, increasing this volumetric flux decreases stimuli effect. This stimulus is once again described by a logarithmic law, given by
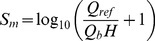
(30)where *Q_ref_* is the largest value of *Q_b_* in the network. In this simulation, the flow rate of parent vessels corresponds to *Q_ref_*.

#### 3.3.4 Vascular adaptation

The total signal for diameter adaptation is represented in the model by the following equation, which remodels the structure of the capillary network
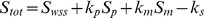
(31)


Here, the parameter *k*_p_ is introduced to indicate the adaptive response sensitivity of vessel diameter to changes in intravascular pressure [22]. *k_m_* is the sensitivity of the adaptive response of the vessel diameter to changes in metabolic state [34].

The vessel has a tendency to reduce in size in the absence of positive growth stimuli. The shrinking tendency, *k*_s_, is applied to show this tendency [22]. Finally, by substituting Equations (27), (28), and (30) into Equation (31), the model for vessel adaptation is given by the following equation:
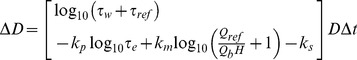
(32)


### Numerical Simulation

Intravascular pressure is numerically calculated at each node by integrating Equations (1) and (8), and then P_b_ in each node (c) is given by
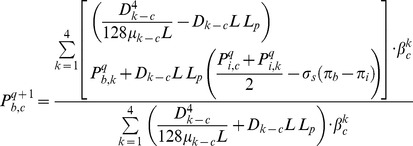
(33)where *q* is the number of iterations. This procedure leads to a set of linear equations for the nodal pressures. These equations can be solved numerically using the Successive Over-Relaxation (SOR) algorithm. Once nodal pressures are known, Equation (7) can be used to calculate the flow rate in each capillary element in turn.

The interstitial pressure is found by solving Equation (15). The iterative numerical method is used to solve the discretized form of Equation (15), given as
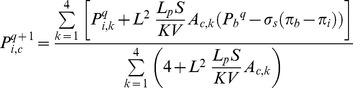
(34)where *q* again is the number of iterations, and *A* is 1 or 0, describing the existence of a blood source. To solve this equation, the SOR algorithm is used.

### 4.1 Algorithm for Calculating Fluid Flow in Capillary Network with Remodeling

**Figure 9:**
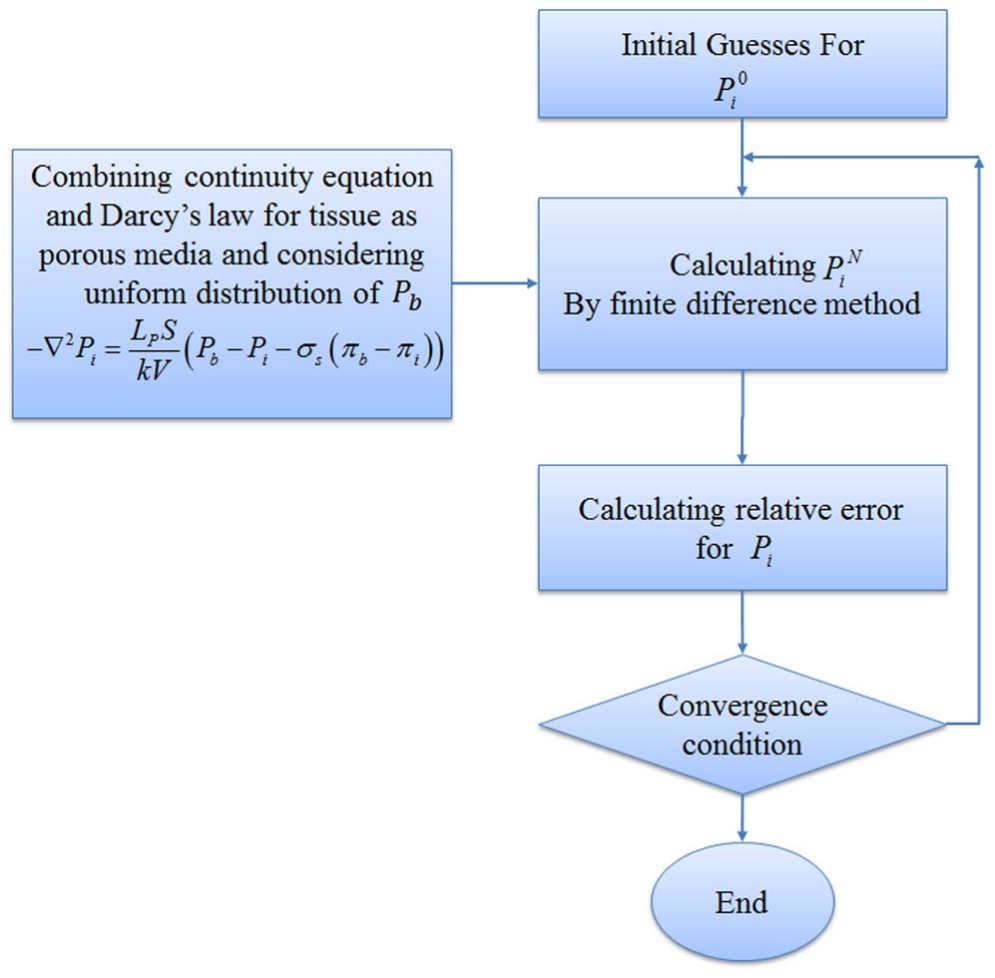
Algorithm for calculating interstitial pressure in tissue without considering capillary network.

**Figure 10:**
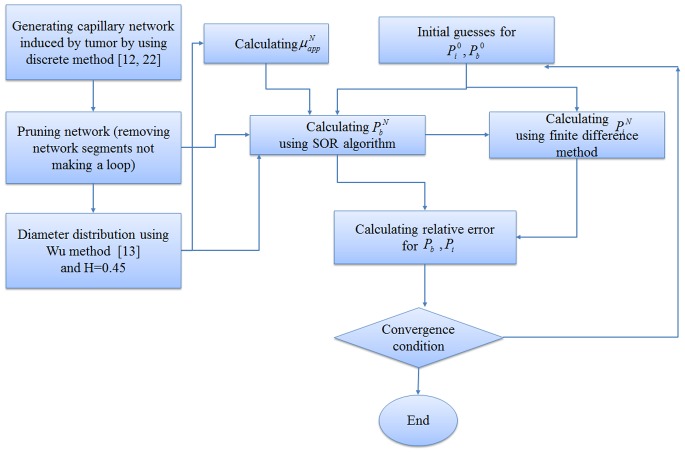
Algorithm for calculating interstitial pressure in tissue and blood flow through capillary network with rigid vessels.

**Figure 11:**
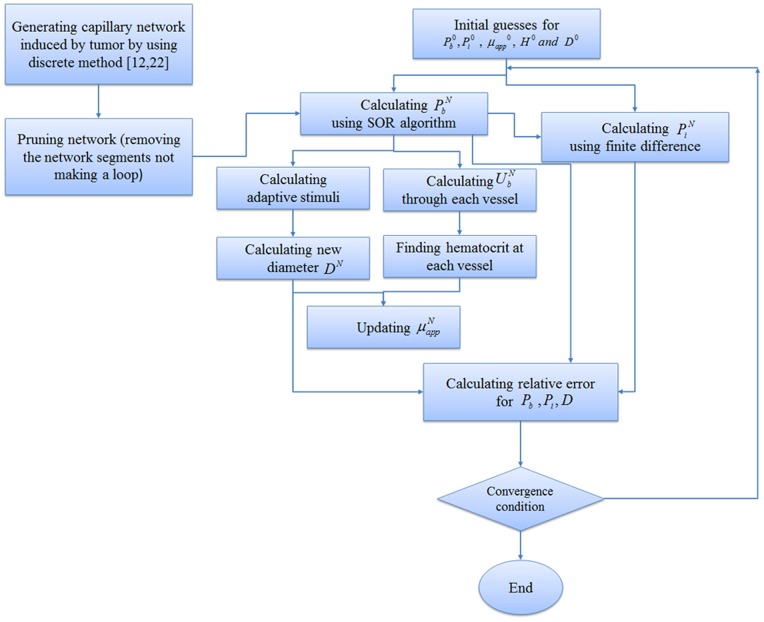
Algorithm for calculating interstitial pressure in tissue and blood flow through capillary network with adaptable vessels and non-continuous behavior of blood.

A systematic flow chart such as one shown by Wong et al. [16], [35] is illustrated for each approach in Figures (9), (10) and (11) to clarify the computational techniques involved in this paper.

The fluid flow calculation in capillary vessels includes a set of non-linear equations. For this reason, an iterative procedure is applied to solve the fluid flow and remodeling equations in a capillary network. The algorithms of three approaches used in this paper for interstitial pressure calculation are shown in Figures (9), (10) and (11). Relative error mentioned in these figures is defined by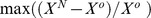, where X can be each of 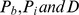 and index *N* is used for a calculated parameter in the current step and *o* is used for a calculated parameter from the previous step.

Figure (9) shows procedures of fluid flow calculation in normal and tumor tissues without a network. Figure (10) shows calculation procedures for the second approach, fluid flow through a capillary network with rigid vessels. For this purpose, the capillary diameter is prescribed using a method presented by Wu et al. [13] and the hematocrit in the vessels is assumed to be constant and equal to 0.45. The viscosity for each segment is calculated based on Equation (18). Figure (11) shows the algorithm of the third approach, model assuming a non-continuous behavior of blood and adaptability of capillary diameters for blood flow calculation through capillary network and fluid flow in tissue. The network pruning mentioned in Figures (10) and (11) is necessary before solving the fluid flow equations. This is achieved by removing the network segments which do not make a loop, i.e. those which have less than two neighbors. This procedure is carried out throughout the entire network repeatedly until all isolated segments are removed.

## Model Parameterization and Simulation Details

### 5.1 Initial Conditions

The 2D domain (shown in Figure 6) considered for the computational simulation studies is a square of length L = 2 mm, and the parent vessel from which the vascular network grows is located at the left edge of the domain. The tumor is located along the right edge of the domain. The equations for tumor-induced capillary growth are solved numerically on a 100×100 (x,y) square grid.


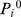
 is initialized to be zero, and 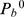 is considered to be 1330 Pa, based on boundary condition explained on section 5.2.2.

### 5.2 Intravascular Flow Model Parameters

In order to simulate the fluid flow in capillaries, several important physical and biological parameters must be first estimated.

#### 5.2.1 Vessel diameter

The initial diameter of each capillary segment is assumed to be 12 µm, and for the parent vessel it is 28 µm, a value that stays constant during the remodeling procedure. During the remodeling process, allowable diameter changes are in the range of 4 µm to 24 µm [36].

#### 5.2.2 Adaptation parameters and phase separation

Parameters used for the adaptation model and phase separation presented in Equations (22) to (24) and (32) are listed in Table (2).in which Q_ref_ is the flow rate in the parent vessel, calculated by Equation (7) assuming D = 28 µm, L = 2 mm, and ΔP = 1950 Pa (15 mmHg) (the pressure drop across the parent vessel), and µ_app_ = 0.0031Pa.s calculated by Equation (18). The plasma viscosity, µ_plasma_ is assumed to be 1.2×10^−3^ Pa.s. The discharge hematocrit H = 0.45 is assumed to stay constant in the parent vessel [22]. The intravascular pressure, P_b_, is an important factor in the vascular remodeling process. In this simulation, inlet and outlet pressures are chosen in the hope of guaranteeing that the average intravascular pressure is around 2660 Pa (20 mmHg), based on the physiological values reported in the literature [7]. Taking into account the physiological condition at microvascular scale results in considering 3325 Pa (25 mmHg) for inlet and 1330 Pa (10 mmHg) for outlet pressures.

**Table 2:** Parameter values used in the adaptation.

Parameter	Value	Reference
τ_ref_ [Pa]	0.103	[22]
Q_ref_ [mm^3^/s]	4.87e-3	Calculated based on network situation
k_p_ [1/s]	0.1	[18]
k_m_ [1/s]	0.07	[18]
k_s_ [1/s]	0.35	[18]
U_cr_	2.5	[30]
ζ	0.5	[30]

### 5.3 Interstitial Flow Model Parameters

The material properties for tumor and normal tissues used for interstitial flow calculation are taken from the simulation study of Soltani et al. [2] and are shown in Table (1).

## Results

As mentioned, three approaches are used for calculating interstitial pressure in a cancerous tissue. First, the results of the first approach introduced in Figure (9) are presented for normal and tumor tissues without a network. Then the results of the second and third approaches, shown in Figures (10) and (11) for two different capillary networks, are presented.

**Figure 12:**
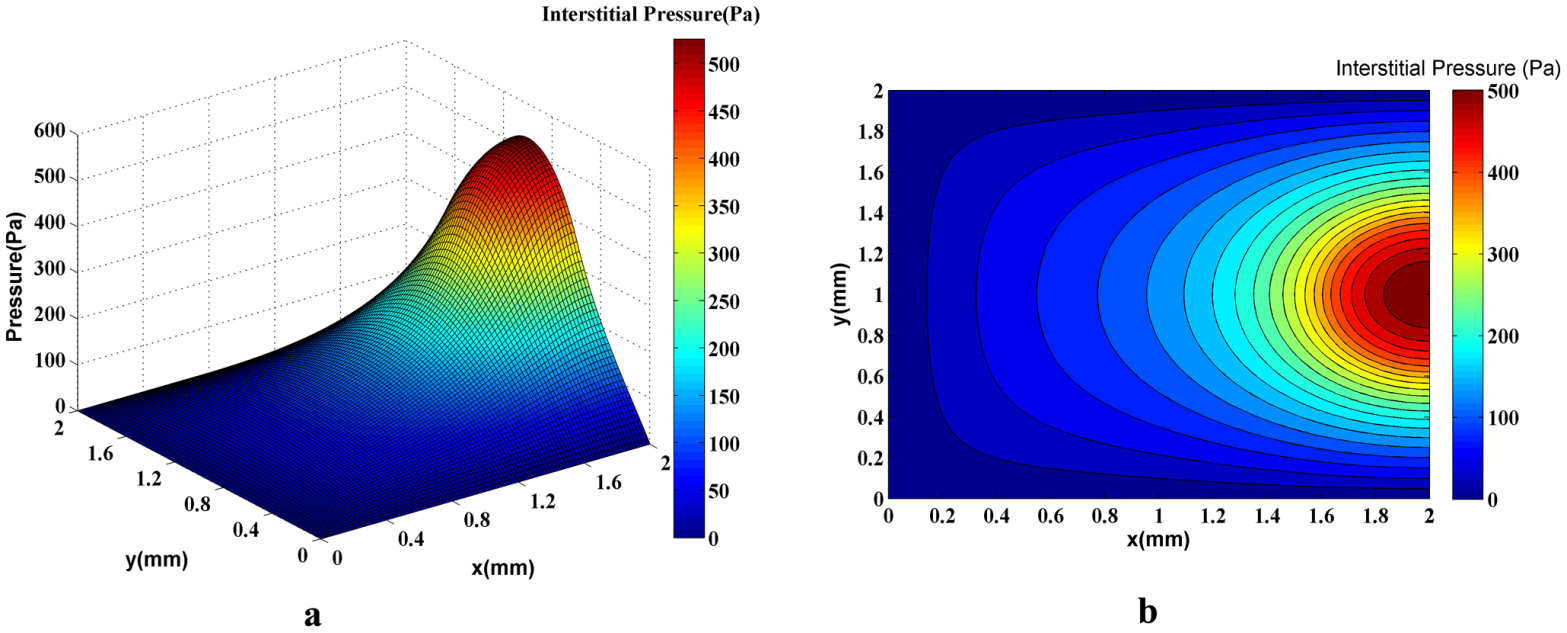
A 3D graph (a) and the contour (b) of interstitial pressure in the computational domain for both normal and tumor tissues in which uniform distribution and constant values for intravascular pressure are assumed. The maximum pressure is around 500Pa in the tumor region.

In order to show the effect of capillary network on the fluid flow in normal and tumor tissues, the tumor’s interstitial fluid flow is simulated without considering such a network. The intravascular pressure, P_b,_ is assumed to have a uniform distribution in the domain and is held constant throughout the simulation. A 3D plot of the interstitial pressure distribution is shown in Figure (12a). Figure (12b) shows the corresponding 2D contour of interstitial pressure. The calculated interstitial pressure of a 0.2-mm radius solid tumor located in a 2 mm×2 mm domain (Figures 12a and 12b), reached a maximum value of approximately 500 Pa.

**Figure 13:**
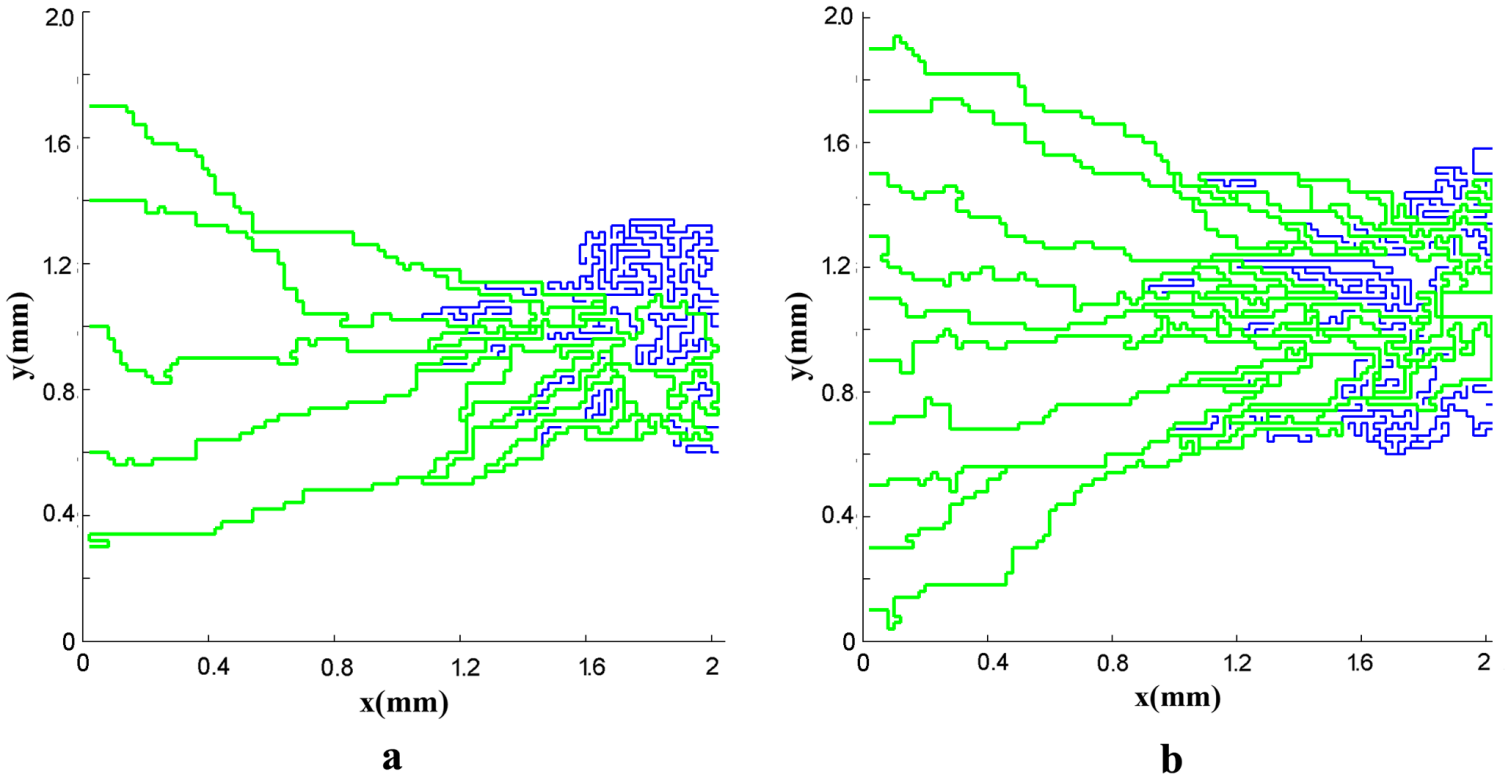
The vascular network after pruning for a network with five (a) and ten (b) endothelial cells in parent vessel. The green lines show the pruned network. The blue lines are killed segments that do not make a loop.

Next, the results of fluid flow in normal and tumor tissues are presented in presence of the capillary network induced by the tumor. The networks shown in Figures (2) and (3) are used for simulating the blood flow. Resulted networks based on discrete method agree well qualitatively with the experiments done in animal corneal models [37], [38] and mathematical model presented by Anderson et al. [12], [39]. The pruned networks are illustrated in Figures (13a) and (13b) for different initial values of endothelial cells.

**Figure 14:**
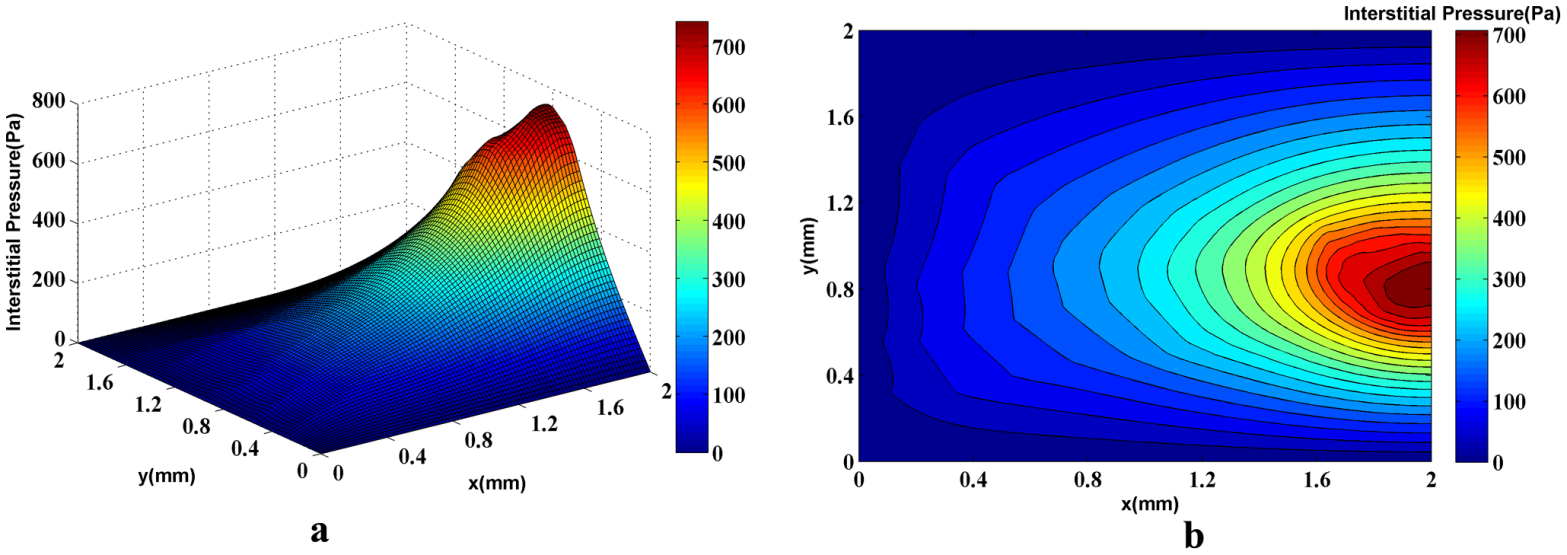
The 3D graph (a) and the contour (b) of interstitial pressure in the computational domain for both normal and tumor tissues for a network with 5 endothelial cells in the parent vessel. The figure is obtained by simulating blood flow through a vascular network found by the discrete sprouting angiogenesis method, with rigid capillaries and continuum properties of blood and coupling by fluid flow in tissue. The maximum pressure is around 700Pa in the tumor region.

**Figure 15:**
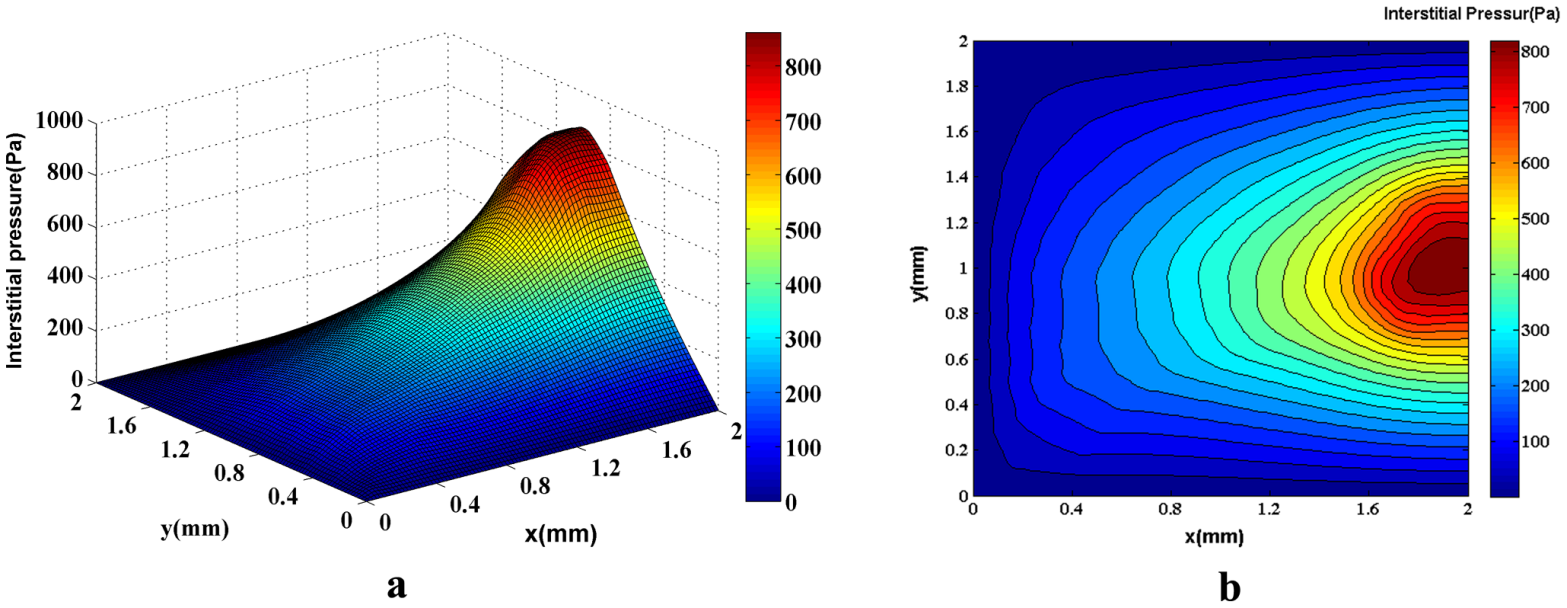
The 3D graph (a) and the contour (b) of interstitial pressure in the computational domain for both normal and tumor tissues for a network with 10 endothelial cells in the parent vessel. The figure is obtained by simulating blood flow through a vascular network found by the discrete sprouting angiogenesis method, with rigid capillaries and continuum properties of blood and coupling by fluid flow in tissue. The maximum pressure is around 800Pa in the tumor region.

The obtained pruned network is used for fluid flow calculation for the second approach based on the algorithm introduced in Figure (10). The 3D graph of interstitial pressure is shown in Figures (14a) and (15a) for different numbers of endothelial cells. The corresponding 2D contour is also illustrated in Figures (14b) and (15b). This method results in higher pressure values in the solid tumor region for both networks. The maximum value of the interstitial pressure was initially higher than 700 Pa for the network with 5 endothelial cells and 800 Pa for the network with 10 endothelial cells.

**Figure 16:**
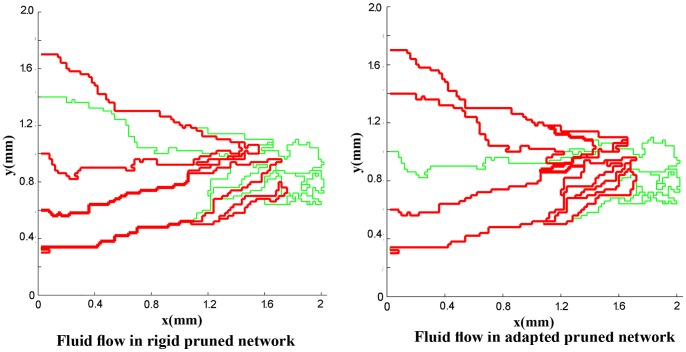
Fluid flow in simulated rigid network and adapted network for network with 5 endothelial cells. In adapted network, some segments eliminated because their diameter is small and they pass very low flow. In both cases, when a segment’s flow rate is less than 0.01 of the maximum flow rate in the network, this segment is pruned.

**Figure 17:**
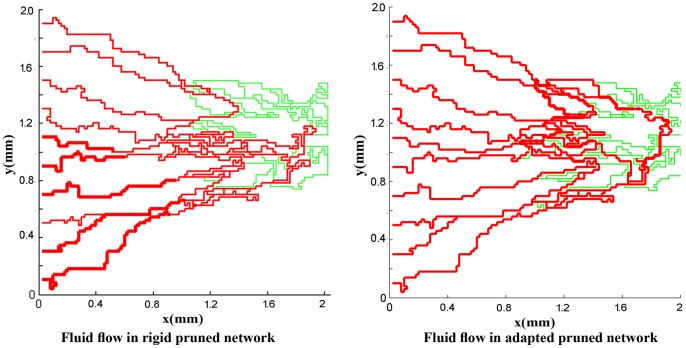
Fluid flow in simulated rigid network and adapted network for network with 10 endothelial cells. In adapted network, some segments eliminated because their diameter is small and they pass very low flow. In both cases, when a segment’s flow rate is less than 0.01 of the maximum flow rate in the network, this segment is pruned.

**Figure 18:**
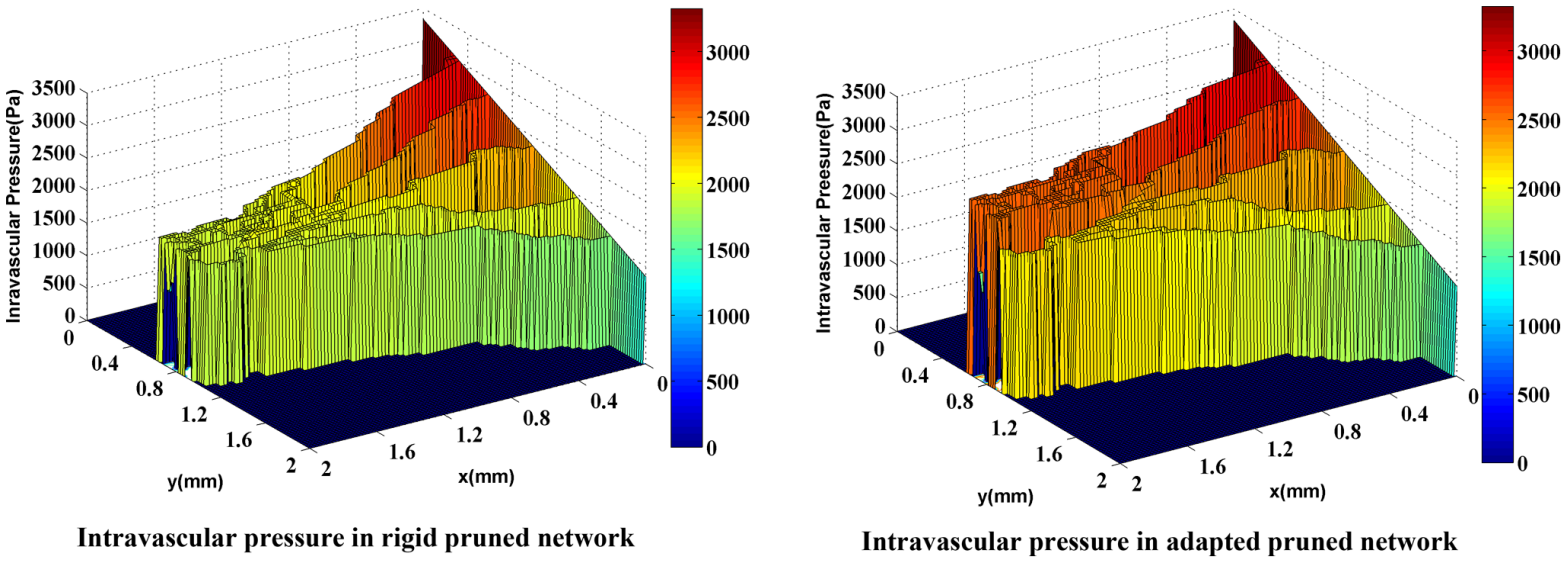
Intravascular pressure in two cases: rigid network and adaptive network for network with 5 endothelial cells. Adaptive network shows higher pressure near the solid tumor. This pressure can affect interstitial flow in this region.

**Figure 19:**
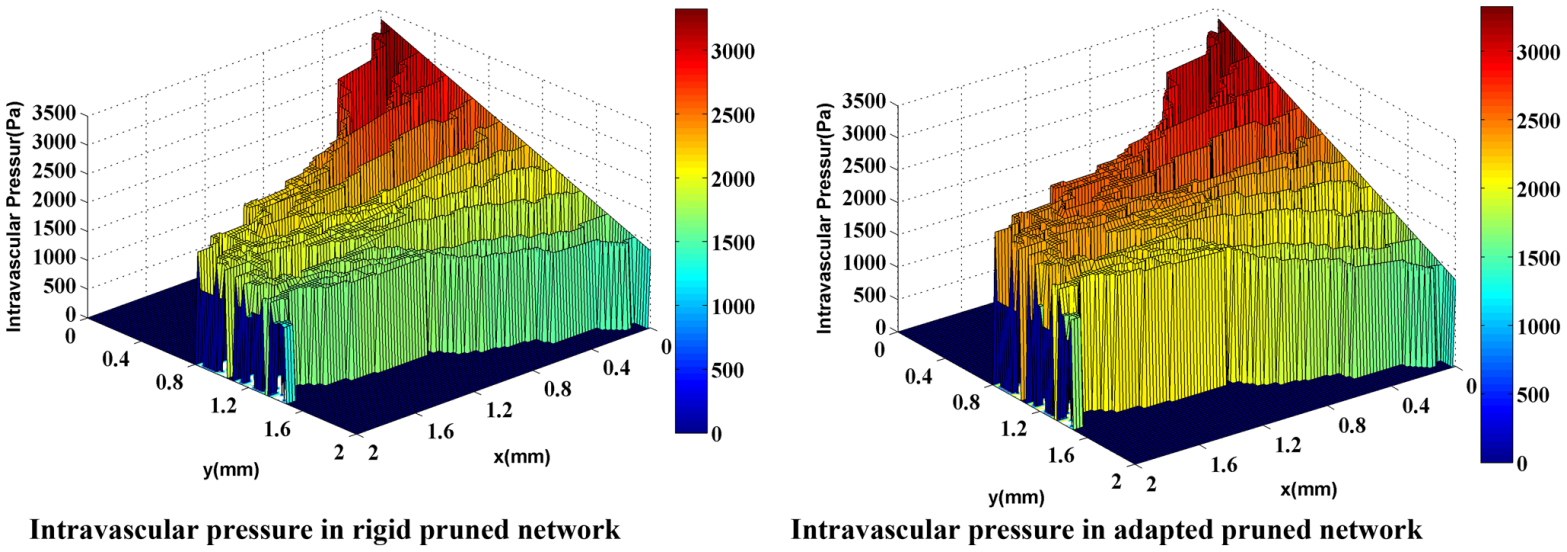
Intravascular pressure in two cases: rigid network and adaptive network for network with 10 endothelial cells. Adaptive network shows higher pressure near the solid tumor. This pressure can affect interstitial flow in this region.

For a more realistic simulation of the system, the effects of non-Newtonian blood rheology, non-uniform distribution of hematocrit in bifurcation due to non-continuous behavior of blood, and remodeling of the network due to adaptability of capillary are coupled to the second approach. The procedure of solution is shown in Figure (11). Figures (16) and (17) show the fluid flow for a network with both adaptive and rigid capillaries, depicting a uniform distribution of flow in the adaptive capillary network. To further illustrate these results, another pruning method was applied to eliminate the capillaries that have a flow rate of less than 1% of the network’s maximum flow rate. In the first approach, many capillaries near the tumor were eliminated; however, in the remodeled network, these capillaries were largely retained. Results for intravascular pressure in the two cases are presented in Figures (18) and (19). The pressure at the right side of domain – the solid tumor region – is higher than that of the rigid network. In fact, based on Starling’s law, the higher intravascular pressure can increase the transvascular flow and slightly affect interstitial flow.

**Figure 20:**
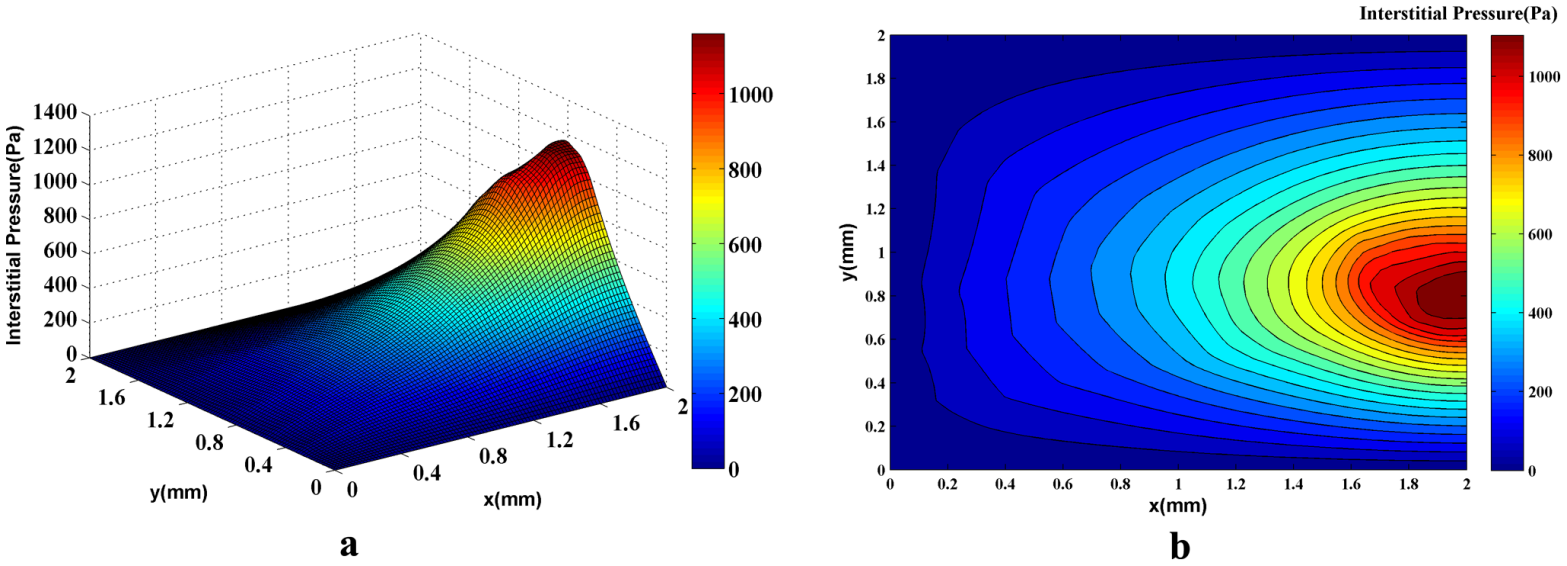
The 3D graph (a) and the contour (b) of interstitial pressure in the computational domain for both normal and tumor tissues for a network with 5 endothelial cells in the parent vessel. Figure is obtained by simulating blood flow through a vascular network found by the discrete sprouting angiogenesis method, with adaptable capillaries and non-Newtonian and non-continuous properties of blood and coupling by fluid flow in tissue. The maximum pressure is above 1100Pa in the tumor region.

**Figure 21:**
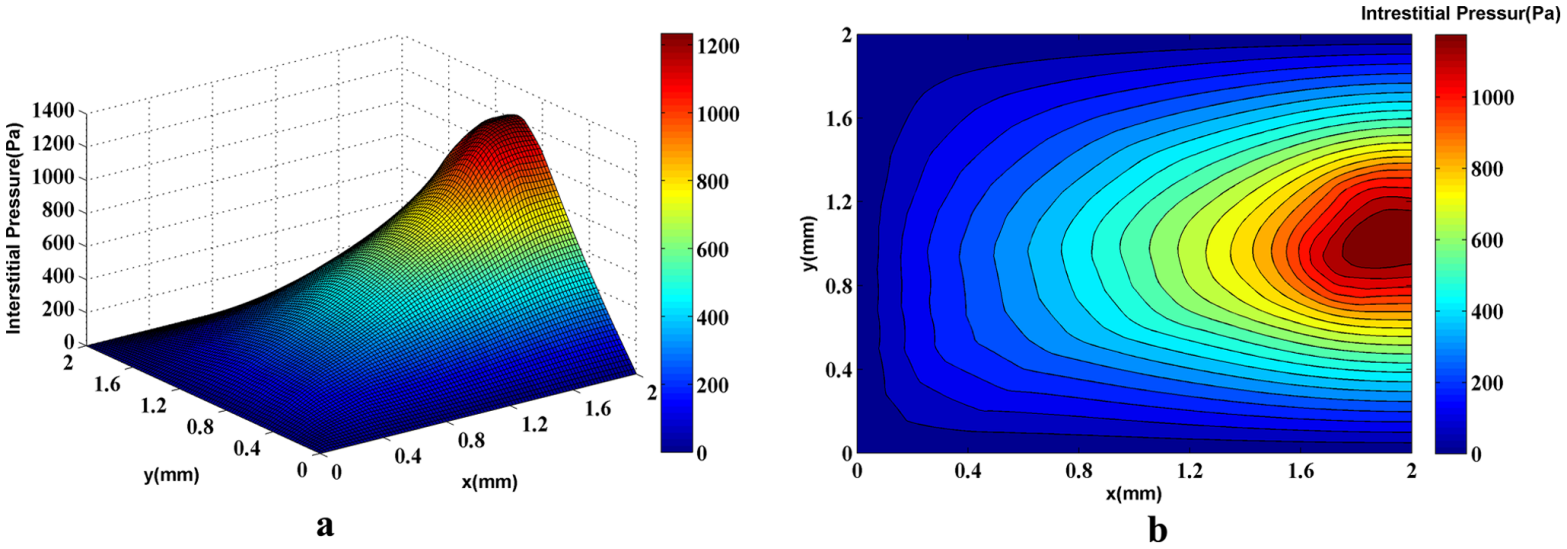
The 3D graph (a) and the contour (b) of interstitial pressure in the computational domain for both normal and tumor tissues for a network with 10 endothelial cells in the parent vessel. Figure is obtained by simulating blood flow through a vascular network found by the discrete sprouting angiogenesis method, with adaptable capillaries and non-Newtonian and non-continuous properties of blood and coupling by fluid flow in tissue. The maximum pressure is above 1200Pa in the tumor region.

Figures (20a) and (21a) show the interstitial pressure in normal and tumor tissues when the adaptation method and phase distribution in the network are also considered (third approach). The corresponding 2D contours are illustrated in Figures (20b) and (21b). Results show higher pressure levels compared to other cases discussed earlier in this study. The calculations predict initial pressure values of around 1100 Pa for the network with 5 endothelial cells and 1200 Pa for the network with 10 endothelial cells.

## Discussion

To accurately predict the efficacy of chemotherapy, researchers must be able to calculate the interstitial and intravascular pressure of tumors with reasonable precision. In many studies, the intravascular pressure is considered to be constant in the fluid flow model which obviously is far from reality. This study simultaneously investigates the fluid flow in a tumor-induced capillary network and the interstitial fluid flow in normal and tumor tissues. For all simulations, a rectangular 2D domain, shown in Figure (6), is considered, in which a circular tumor is located on one side and the parent vessel is located on the opposite side.

The results based on first approach (Figure 9) or simulation of fluid flow in tissue without network showed that the interstitial pressure is at its maximum at the center of the tumor and diminishes towards the periphery. In tumor tissue, capillaries are more permeable. This high permeability results in more extravascular flow based on Starling’s law. Contrary to normal tissue, lack of lymphatic system and above mentioned high extravascular flow result in the high level of interstitial pressure in tumor tissue. The high elevation of interstitial pressure is observed in previous works [2], [10], Jain et al. [8], and in the experimental results of Arifin et al. [40] and Huber et al. [41].

Results show that the number of endothelial cells does not significantly affect the intravascular pressure and fluid flow distribution as the final structures of the networks are almost independent of the endothelial cell numbers. As described earlier, two approaches – rigid and adaptable capillary networks – were investigated. The flow distribution, specially for remodeled networks (Figures 16b and 17b), is similar to flow distribution observed in the literature [18], [12]. The results show irregular blood vessels which is similar to the results visualized by Heine et al. [42] by DiI labeling method.

The comparison of blood flow illustrated in Figures (16) and (17) for the two networks show that the blood flow in the capillary network for the remodeled network has a more logical distribution than those with rigid capillaries; it is due to considering vessels’ adaptation to metabolic and hemodynamic stimuli. Considering metabolic stimuli causes blood flow in the vessels far from parent vessels. Hemodynamic stimulus, especially intravascular pressure, prevents shunt fluid path [34].

The reasonable distribution of blood flow in the remodeled network, which leads to a declined blood-flow resistance far from the parent vessel, increases the value of intravascular pressure in the network especially near the tumor, as shown in Figures (18) and (19). The existing blood vessels are immature [42]. Immature blood vessels together with the elevated intravascular pressure lead to an increase in the transvascular flow rate, based on the Starling’ law which subsequently affects the interstitial pressure. These results are in a good agreement with experimental results presented by Chauhan et al. [43]. Also, intravascular pressure distributions show that considering uniform distribution of source terms with constant intravascular pressure is not a reasonable assumption as may result in totally different values for intravascular pressure.

Results of interstitial pressure distribution for both networks and two approaches are shown in Figures (14a), (15a), (20a), and (21a). The two approaches to blood flow modeling through capillaries considered in the fluid flow simulation for both networks show significantly different results than those for the case without a network (i.e. uniform distribution of source terms with constant intravascular pressure [2], [10]). Results of constant vessel size (Figures 14a and 15a) show an approximately 1.5 times increase on the maximum pressure resulting from uniform distribution of source terms with constant intravascular pressure. The differences between the two networks are due to the higher density of vessels close to the tumor region. The maximum value of interstitial pressure has a good agreement with the numerical simulation of a brain tumor reported by Tan et al. [44] in which the geometry is reconstructed from the magnetic resonance images of a primitive neuroectoderma tumor. Generally, in brain, the capillaries have more rigid characteristic than other capillaries. The simulated pressure is around 900 Pa in their work. Results are also similar to the simulation results of Zhao et al. [45].

The interstitial pressure calculations based on the third approach (Figures 20a and 21a) – an adaptable capillary to blood flow through capillaries– show pressure levels approximately 1.5 times greater than that of the rigid network and more than 2 times greater than that of the uniform distribution of source terms with constant intravascular pressure. Measurements of 6 different HT29 tumors by Heine et al. [42] show a mean interstitial pressure of 1190 Pa that support simulation results in the present study with value around 1200 Pa shown in Figures (20a) and (21a).

The interstitial pressure for normal and tumor tissues without a network shows a regular distribution (Figure 12b), but the evaluation of contours in Figures (14b), (15b), (20b), and (21b) indicates that the regular distribution of pressure is altered due to irregular source terms of mass flow to the tissue. The recent reports published by of Chauhan et al. [43] and Arifin et al. [46] show this phenomena. They validated the used numerical method by some *in* *vivo* images.

### Conclusions

One of the main results of this study is that contradictory to previous works in the interstitial flow, the intravascular pressure is not fixed and will be updated during the solution.

The interstitial pressure in the case of tumor-induced capillary network was observed to be higher than what was shown in the case of no network, as well as those reported by our group where a uniform distribution of source terms with constant intravascular pressure was assumed. The maximum value of interstitial pressure has a good agreement with the numerical simulation of a brain tumor reported by Tan et al. [44].

The method with adaptive capillary network showed the highest interstitial pressure in the tumor region. This method assumed the capillary diameter to be an adaptable function of hemodynamic and metabolic stimuli. Considering the adaptability of vessels and non-continuous behavior of blood leads to a more-uniform blood flow distribution compared to the case of a rigid capillary network. In the method with adaptive capillary network, capillaries far from a parent vessel experience a non-negligible flow, whereas in the case of a rigid capillary network, many of these capillaries are eliminated because of their low flow rates. The results of maximum interstitial pressure are compared with recent experimental data for HT29 tumor. The value of simulation is very close to the experimental data. The high interstitial pressure in the tumor region, which along with the low transvascular flow due to low intravascular pressure in the heterogeneous capillary network near the tumor region, plays a significant role in non-uniform distribution of drug delivery to a solid tumor.
